# Osteoperiosteal Cylindrical Iliac Bone Graft and Minced Cartilage as an Osteochondral Autograft for a Large Osteochondral Lesion

**DOI:** 10.1016/j.eats.2024.103135

**Published:** 2024-07-29

**Authors:** Murat Bozkurt, Daria Nayda, Ali Şahin, Kadir Yavaşoğlı, Enejd Veizi

**Affiliations:** a Department of Orthopedics and Traumatology, Ankara Acıbadem Hospital, Ankara, Turkey; b Burdenko Main Military Clinical Hospital, Moscow, Russia; c Department of Orthopedics and Traumatology, Ankara Etlik City Hospital, Ankara, Turkey; d Department of Orthopedics and Traumatology, Ankara Yıldırım Beyazıt University, Ankara Bilkent City Hospital, Ankara, Turkey

## Abstract

Mosaicplasty is a relatively challenging procedure used in the management of focal osteochondral lesions of the joints. Donor-site morbidity is still the main concern after mosaicplasty because it entails the harvesting of an osteochondral autograft from an otherwise healthy region to be impacted later on the weight-bearing damaged site. We describe a possible alternative to conventional mosaicplasty with subchondral bone support harvested from the iliac crest as an osteoperiosteal autograft and covered with a minced cartilage layer.

Previous studies have documented favorable outcomes after arthroscopic microfracture and debridement in the management of osteochondral lesions characterized by relatively shallow subchondral defects.^1^, ^2^, ^3^ However, in cases of larger and deeper subchondral defects or in the presence of subchondral cysts, restoration of the subchondral bone defect alongside the articular cartilage may be necessary.^4^ The osteochondral autograft transfer system (OATS), involving the transplantation of cylindrical autografts, is commonly used in such scenarios, yet concerns persist regarding donor-site morbidity.^5^ Concurrently, the OATS procedure (often referred to as “mosaicplasty”) can prove unsuitable for large osteochondral lesions.^6^ Consequently, alongside the standard adoption of OATS, osteochondral allografts and minced cartilage techniques have garnered increasing favor in clinical practice.^7^^,^^8^

The aim of our technique is to circumvent the limitations associated with the OATS by using cylindrical subchondral bone obtained from the iliac crest and impacted as subchondral support, followed by coverage with minced cartilage and transplantation to the recipient site with precise dimensions.

### Preoperative Considerations and Indications

The described technique is indicated for large chondral and osteochondral lesions, but there is not yet a consensus on the optimal size of the lesion. The main purpose is to retrieve the cells from their domains to repopulate the new lesion and bridge the defective cartilage with a new matrix.^9^ Physical examination usually reveals pronounced pain in the affected compartment of the joint, accompanied by instances of locking during movement. Computed tomography scans are generally used to reveal the osseous defect within the joint, a finding usually consistent with magnetic resonance imaging results generally depicting full-thickness osteochondral lesions.

## Surgical Technique

**Fig 1:**
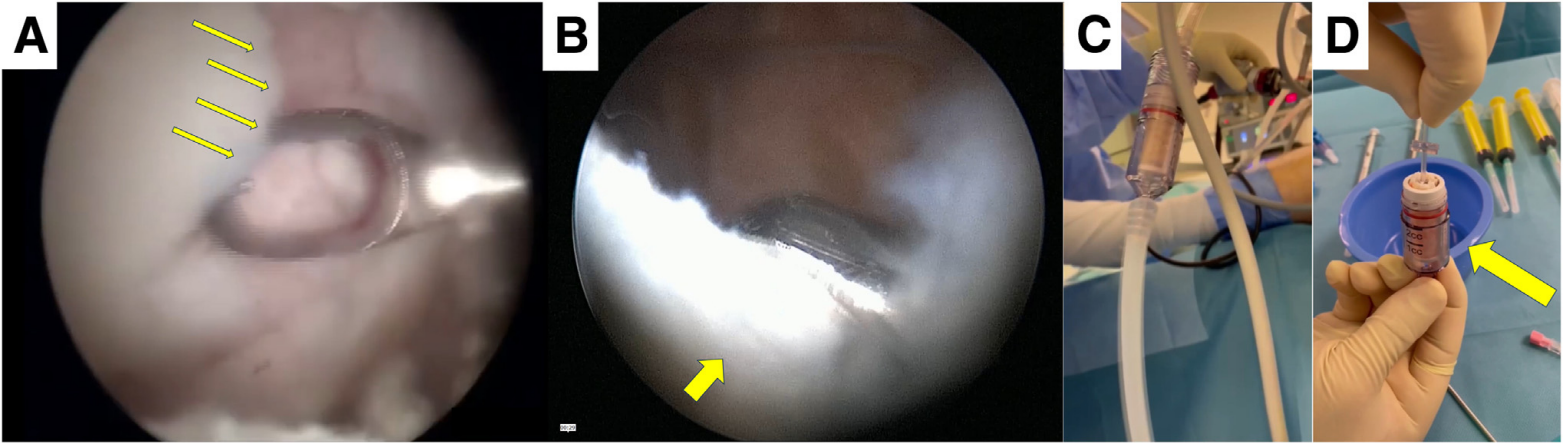
(A) Osteochondral lesion on the medial femoral condyle of the right knee as viewed from the anterolateral portal. The lesion’s margins are debrided with a curette (arrows) from the anteromedial portal. (B) Healthy cartilage tissue is harvested from the non–weight-bearing area of the lateral condyle (arrow) with a shaver inserted from the anterolateral portal. (C) The harvested tissue is collected with an external graft harvester. (D) The harvested tissue is separated (arrow) for subsequent use.

**Fig 2:**
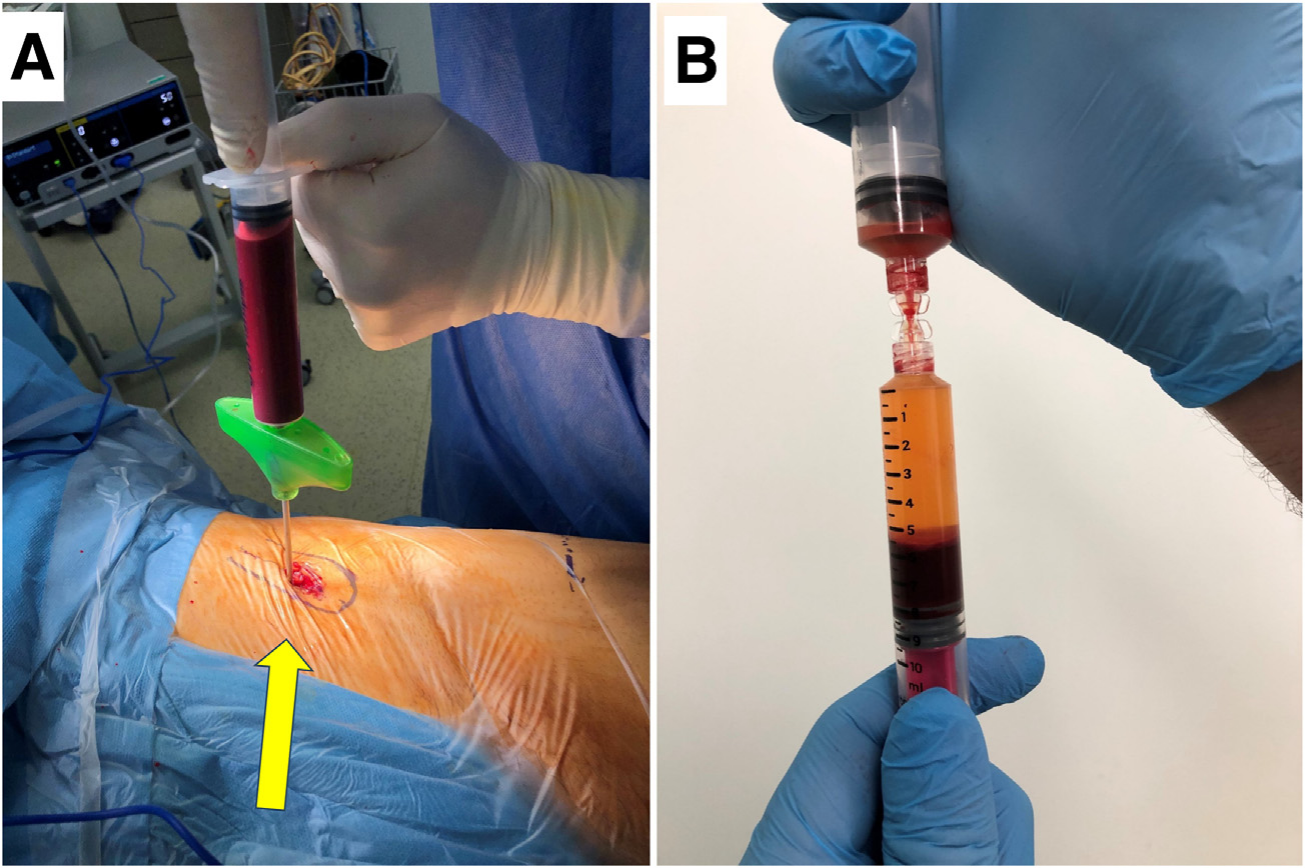
A bone marrow aspiration needle is inserted on the ipsilateral iliac crest (A, arrow) (left side, patient supine, left cephalad), and 60 mL of bone marrow is aspirated and then concentrated into bone marrow aspirate concentrate (B).

**Fig 3:**
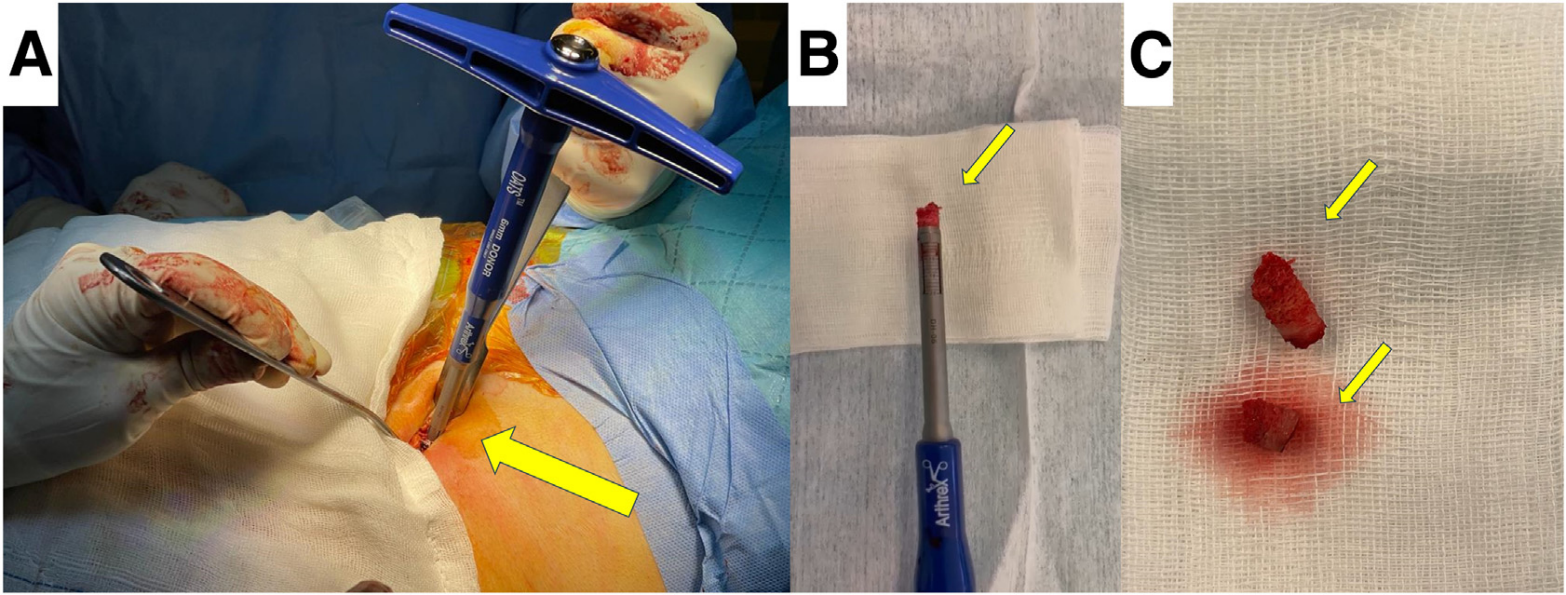
(A) At the same spot of aspiration, a small incision is performed and the iliac crest’s bone (arrow) is exposed (patient supine, left iliac crest, right cephalad). (B, C) By use of an autologous osteochondral graft harvester (arrow), 2 cylindrical osteoperiosteal autografts (arrows) with a length of at least 15 mm are obtained and stored for later use.

The surgical procedure is meticulously demonstrated in Video 1. Initial steps involve peripheral blood collection for autologous conditioned serum (ACP; Arthrex) before anesthesia to avoid mixing with toxic agents. Standard knee arthroscopy is then conducted through the anteromedial and anterolateral portals. Intra-articular inspection using a probe reveals a conspicuous osteochondral lesion situated on the medial femoral condyle, whereas no significant pathology is detected in the other knee compartments (Fig 1A). The area is debrided, and the peripheral cartilage of the lesion, together with portions of a non–weight-bearing surface of the knee, is specifically collected with a shaver and a GraftNet (Arthrex) (Fig 1 B-D). Subsequently, a longitudinal medial mini-incision is made at the level of the lesion, followed by dissection and incision of the retinaculum and capsule. The joint is exposed, and the margins of the lesion are carefully delineated and measured. Approximately 60 mL of bone marrow is aspirated with a bone marrow aspiration needle, followed by concentration using the BMAC2-60-01 Procedure Pack of the Angel BMAC Cellular Therapy System (Arthrex) (Fig 2). At the same aspiration point, a small incision is performed and the local iliac crest is exposed (Fig 3A). With the help of an osteochondral graft harvester, 2 cylindrical osteoperiosteal autografts are obtained, each 15 mm in length. These grafts are extruded and trimmed to fit the prepared host site, calculating for minimal impaction (Fig 3 B and C). Subsequently, a small medial parapatellar incision is performed on the knee, and the lesion is exposed. Two size-matched tunnels with a depth of 15 mm are drilled. The 2 cylindrical osteoperiosteal iliac bone grafts are over-impacted on the respective drilled tunnels to avoid increased peak pressures and surface mismatch. A microfracture procedure is then performed in the surrounding area of the host region to increase blood supply.

**Fig 4:**
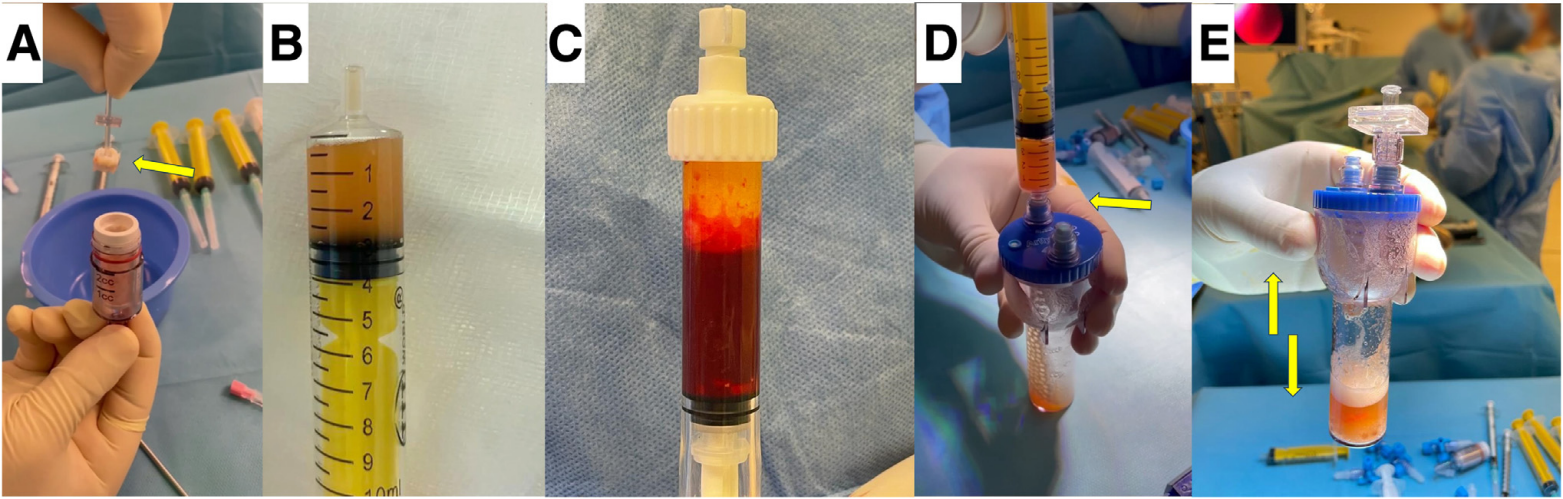
On the side table, the harvested healthy chondral tissue (A, arrow), the preoperatively prepared conditioned serum (B), and the bone marrow aspirate concentrate (C) are injected into the Thrombinator system (D, arrow) and are shaken together (arrows) to obtain a homogeneous mixture (E).

**Fig 5:**
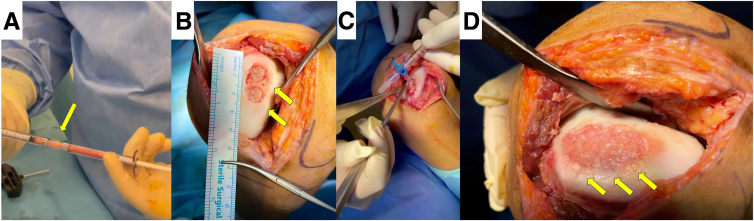
(A) The ultimate mixture from the Thrombinator system is further mixed with the double-syringe system (arrow) until a putty solution of minced cartilage is obtained. (B) The previously obtained autologous osteoperiosteal grafts are impacted into the previously drilled medial femoral condyle (arrows) (left flexed knee, medial parapatellar incision, superior cephalad). (C) The minced cartilage obtained from the double-syringe system is spread over the damaged area. (D) The surface is flattened and contoured accordingly (arrows). After a minimum of 5 minutes, the knee is first mobilized and then closed in standard fashion.

On a separate table, the autologous conditioned plasma, bone marrow aspirate concentrate, and healthy cartilage tissue obtained from the GraftNet (Fig 4 A-C) are mixed in the Thrombinator system (Arthrex) to obtain a putty solution of minced cartilage (Fig 4 D and E). The double-syringe system is then used to further consolidate the mixture (Fig 5A). Finally, the putty solution of minced cartilage from the double syringe is spread as the last layer on the damaged region of the condyle, and a congruent surface is achieved (Fig 5 B and C). After waiting at least 5 minutes for consolidation (Fig 5D), the knee joint is mobilized and then closed in routine fashion (Tables 1 and 2). A bandage is applied, but no Hemovac (Zimmer Biomet) is applied. The iliac crest is similarly closed in standard fashion.

**Table 1:** Pearls and Pitfalls of the Technique

Pearls
The conditioned serum should be obtained before anesthesia begins to avoid mixture with possible toxins. Bone marrow aspiration can be performed after the iliac crest bone is exposed. The damaged area should be drilled to a depth matching the obtained subchondral grafts or slightly over-drilled.
Pitfalls
Failure to impact the subchondral grafts into the damaged area and residual protrusion can create high peak pressures on the surgical site, leading to constant pain. After flattening or over-impaction of the subchondral grafts, failure to flatten or meticulously contour the condyle can lead to increased peak pressure areas.

**Table 2:** Advantages and Limitations of the Technique

Advantages
The procedure does not create extra intra-articular cartilage damage because it is not based on autologous osteochondral transplantation. Because no additional healthy cartilage and subchondral bone are violated within the joint, the procedure is less painful than a standard mosaicplasty.
Limitations
General anesthesia is frequently required. An additional potential painful autograft site (iliac crest) is required. Long-term studies to show a significant clinical advantage are still lacking.

### Rehabilitation

On the initial postoperative day, the patient receives guidance from a physical therapist to engage in a series of hip, knee, and ankle exercises while in bed. These exercises comprise both passive and active range-of-motion exercises, with each session lasting 15 minutes and being performed 3 times daily. Weight bearing on the affected limb is strictly prohibited for a duration of 6 weeks after surgery, with partial weight bearing permitted thereafter. Full weight bearing is sanctioned at the 8-week mark after surgery.

## Discussion

Numerous methods have been documented for addressing osteochondral lesions of the knee.^4^^,^^8^ The treatment approach for such lesions hinges on several crucial factors, including the lesion’s size, depth, and localization, as well as the patient’s age and activity level.^1^^,^^3^ Although fixation may appear promising as an early intervention, particularly for adolescents and young adults, managing sizable osteochondral lesions poses a formidable challenge overall.^4^

In the process of determining treatment strategies, it is imperative to assess the structural integrity of the subchondral bone.^10^^,^^11^ Despite the formation of fibrocartilage, concurrent regeneration of subchondral bone might not occur after the implementation of marrow-stimulating techniques, particularly in cases in which the quality of the subchondral bone has been compromised.^11^^,^^12^ Consequently, the subchondral bone may fail to provide a robust scaffold to support the overlying cartilage adequately. For extensive lesions, bolstering the subchondral bone constitutes the initial step in treatment.^13^

Various techniques and recommendations for grafting can be found in the literature.^5^^,^^6^^,^^14^^,^^15^ Although mosaicplasty presents an attractive approach, wherein both hyaline cartilage and bone are transplanted simultaneously, concerns regarding donor-site morbidity pose significant risks, especially in cases involving large lesions.^5^ Matrix autologous chondrocyte implantation typically involves a 2-stage process and is often combined with bone grafting.^16^ However, these procedures are demanding for patients and entail substantial costs. Osteochondral allografts have emerged as a viable alternative in such scenarios but are encumbered by their own limitations, including issues of availability, high expenses, and complex surgical procedures.^6^^,^^17^ Hu et al.^12^ noted that osteoperiosteal cylindrical grafts harvested from the iliac crest exhibit limited potential for chondrogenesis owing to the absence of a cambium layer in the periosteum. Hence, we advocate the use of osteoperiosteal cylinders of subchondral bone overlaid with a cellular scaffold, as described in this article, to stimulate more robust chondrogenesis.^12^ Additionally, literature studies have delineated techniques using osteoperiosteal cylindrical grafts in conjunction with cell-free or cellular scaffolds.^15^

Minced cartilage has experienced a resurgence in popularity in recent years, primarily owing to advancements in arthroscopic cartilage harvesting techniques, which offer facile biological application.^7^^,^^15^ By minimizing trauma during cartilage removal from the donor site, these methods mitigate the risk of donor-site morbidity. The appeal of minced cartilage lies in its capacity to be harvested from non–weight-bearing areas of the knee joint, its provision of hyaline cartilage, and the favorable outcomes documented in previous studies. Leveraging this approach, we capitalize on the established efficacy of subchondral bone replacement while using osteoperiosteal grafting to promote robust chondrogenesis. Moreover, minced cartilage particles can be securely affixed to scaffolds, ensuring both stable fixation and streamlined rehabilitation protocols.

The methodology delineated herein appears to represent a straightforward, reliable, efficacious, and economically viable approach for managing significant osteochondral lesions of the knee. Nonetheless, to prove this hypothesis, clinical outcomes derived from case series involving patients treated with this technique, along with comparative studies, are imperative.

## Disclosures

All authors (M.B., D.N., A.Ş., K.Y., E.V.) declare that they have no known competing financial interests or personal relationships that could have appeared to influence the work reported in this paper.

## Supplementary Data

Video 1 Surgical technique for osteoperiosteal cylindrical iliac bone graft and minced cartilage as osteochondral autograft for large osteochondral lesion. The patient is placed supine on the surgical table with the ipsilateral knee and iliac crest prepared and draped. Before anesthesia administration begins, peripheral blood is obtained for autologous conditioned plasma. A diagnostic arthroscopy follows, and the lesion is visualized and debrided. By use of the GraftNet system, healthy cartilage tissue is harvested from the non–weight-bearing area of the lateral condyle. Next, approximately 60 mL of bone marrow is aspirated from the iliac crest and concentrated into bone marrow aspirate concentrate. At the same aspiration point, a small incision is performed, and the local iliac crest is exposed. With the help of an osteochondral graft harvester, 2 cylindrical osteoperiosteal autografts are obtained, each 15 mm in length. Subsequently, a small medial parapatellar incision is performed on the knee, and the lesion is exposed. Two size-matched tunnels with a depth of 15 mm are drilled. On a separate table, the autologous conditioned plasma, bone marrow aspirate concentrate, and healthy cartilage tissue obtained from the GraftNet are mixed in the Thrombinator system to obtain a putty solution of minced cartilage. The double-syringe system is then used to further consolidate the mixture. On the open knee joint, the 2 cylindrical osteoperiosteal iliac bone grafts are over-impacted on the respective drilled tunnels to avoid increased peak pressures and surface mismatch. Finally, the putty solution of minced cartilage from the double syringe is spread as the last layer on the damaged region of the condyle, and a congruent surface is achieved. After waiting at least 5 minutes for consolidation, the knee joint is mobilized and then closed in routine fashion. A bandage is applied, but no Hemovac is used. The iliac crest is similarly closed in standard fashion.
